# Suspicion for Sarcoma: Clinical Presentation, Multi-Modality Imaging Evaluation, and Ultrasound Artificial Intelligence-Based Decision Support

**DOI:** 10.3390/cancers17223626

**Published:** 2025-11-11

**Authors:** Nikki A. Mehran, Emily Rooney, Harsh Shah, Tamar Gomolin, Nebras Zeizafoun, Dayna Williams, Laurie R. Margolies, Christine Chen

**Affiliations:** 1 Department of Diagnostic, Molecular and Interventional Radiology, Icahn School of Medicine at Mount Sinai, New York, NY 10029, USA; nikki.mehran@mountsinai.org (N.A.M.); emily.rooney@mountsinai.org (E.R.); harsh.shah@mountsinai.org (H.S.); dayna.williams@mountsinai.org (D.W.); 2 Department of Pathology, Molecular and Cell Based Medicine, Icahn School of Medicine at Mount Sinai, New York, NY 10029, USA; tamar.gomolin@mountsinai.org (T.G.); nebras.zeizafoun@mountsinai.org (N.Z.)

**Keywords:** breast sarcoma, primary breast sarcoma, secondary breast sarcoma, ultrasound, mammography, MRI, artificial intelligence, breast cancer, decision support

## Abstract

**Simple Summary:**

Breast sarcomas are rare and aggressive. Our study aims to better characterize the clinical presentation, histology, and imaging features of breast sarcomas on mammography, ultrasound, and MRI, in addition to analyzing the effectiveness of ultrasound AI decision support (DS) in detecting breast sarcomas. A retrospective review from 2008–2024 yielded 18 patients with histologically proven breast sarcomas with imaging available for review. Mammography was available for 13 lesions, ultrasound for 19 lesions, and MRI for 9 lesions. The most common presentation of breast sarcoma was as an irregular-shaped mass with non-circumscribed margins on mammography, ultrasound, and MRI, the latter with heterogenous enhancement. Ultrasound AI DS accurately identified 15 out of 16 (93.8%) breast sarcoma lesions seen on ultrasound as suspicious. Awareness of how breast sarcomas can present across imaging modalities while using AI DS as an aid may help radiologists in making the correct diagnosis of this rare and aggressive disease.

**Abstract:**

**Background/Objective**: This study aims to better characterize the clinical presentation, histology, and imaging features of breast sarcomas on mammography, ultrasound, and MRI, in addition to analyzing the effectiveness of AI DS in detecting breast sarcomas. **Methods**: A retrospective review from 2008–2024 yielded 18 patients with histologically proven breast sarcomas with imaging available for review. Mammography was available for 13 lesions, ultrasound for 19 lesions, and MRI for 9 lesions. Imaging features were classified according to the BI-RADS 5th edition lexicon. Images were reviewed by two radiologists, and consensus was obtained regarding imaging features. AI DS was retrospectively applied to the breast masses identified on ultrasound. Data analysis was performed using descriptive statistics. **Results**: 17 females and 1 male were included in this study. Mammographic findings varied from solitary masses (3/13 [23.1%]), asymmetries (3/13 [23.1%]), architectural distortion (1/13 [7.7%]), skin thickening (3/13 [23.1%]), focal asymmetry with calcifications (1/13 [7.7%]), or no suspicious findings (2/13 [15.4%]). Sonography often revealed masses with an irregular shape (13/16 [81.2%]), non-circumscribed margins (15/16 [93.7%]), hypoechoic echo pattern (10/16 [62.5%]), and vascular flow (12/16 [75%]). MRI showed heterogeneously enhancing masses (6/9 [66.7%]) or isolated skin enhancement (3/9 [33.3%]). AI DS analyzed 16 masses on ultrasound and identified 15 (93.8%) as suspicious. **Conclusions**: Breast sarcomas had a variable appearance on breast imaging, ranging from a solitary mass to isolated skin findings. Awareness of how breast sarcomas can present across imaging modalities while using AI DS as an aid may help radiologists in making the correct diagnosis of this rare and aggressive disease.

## 1. Introduction

Breast sarcomas constitute a heterogeneous group of nonepithelial tumors arising from the mesenchymal tissue of the breast, representing less than 5% of all soft tissue sarcomas [1,2,3] and less than 1% of all breast malignant neoplasms [4,5,6,7]. Common histopathological diagnoses include fibrosarcoma, angiosarcoma, and pleomorphic sarcoma, with angiosarcoma being the most common [8,9,10]. Sarcomatous tumors enlarge rapidly and metastasize hematogenously [8,9]. The disease is highly aggressive with a 10-year mortality rate of 40% [11]. Breast sarcomas are divided into two categories: primary breast sarcoma (PBS) and secondary breast sarcoma (SBS) [12]. PBS arises de novo with no known triggering event, whereas SBS is caused by exposure to external factors such as previous radiation therapy and chronic lymphedema [13,14,15,16]. Breast sarcoma development is also associated with environmental exposures such as alkylating agents and vinyl chloride, as well as genetic factors including Li–Fraumeni syndrome (TP53 mutation) [13,17,18].

Both PBS and SBS are rare with an estimated annual incidence of 4.6 cases per million women [19]. The literature has described PBS to have an estimated 107 median survival months, while SBS has an average of 45 median survival months, demonstrating the aggressive nature of both subtypes [20]. In addition to the rarity of the disease, the difficulty of diagnosing breast sarcomas is further exacerbated by a nonspecific clinical presentation, sometimes lacking a palpable lump and only presenting with a rash-like appearance [8]. As such, familiarity with the clinical and radiologic findings characteristic of breast sarcoma is paramount for a timely diagnosis.

Recent advancements in artificial intelligence, particularly deep learning (DL), have significantly impacted medical imaging and diagnostic workflows. DL has been applied to various applications, including multi-cancer detection using transfer-learning-enhanced CNNs, low-complexity neural networks for tumor anomaly detection and brain tumor classification, and models that incorporate uncertainty quantification to enhance classification robustness. These works underscore the broad utility of DL across different medical imaging tasks and motivate the continued development and evaluation of AI tools—such as the one employed in our study—for improving diagnostic accuracy and clinical decision-making [21,22,23,24].

Advances in artificial intelligence (AI) have led to the development of various AI systems to assist radiologists in identifying and diagnosing breast lesions. One such system is KOIOS ultrasound artificial intelligence (AI) decision-making support (DS), which analyzes breast ultrasound (US) images by generating a likelihood of malignancy for a user-selected region of interest (ROI) that contains a breast lesion [25]. KOIOS has been shown to improve the diagnostic accuracy of sonographic breast lesions and reduce inter- and intra-observer variability [26,27,28]. Although AI decision-making support has become increasingly popular, there is currently a gap in the literature on how this technology performs when analyzing breast sarcomas [29].

Our study aimed to better characterize the clinical presentation and imaging features of breast sarcomas through mammography, ultrasound, and breast MRI for histologically proven cases. We also included pregnant patients, a male patient, and potentially unusual imaging features such as a lesion that mimics a cyst in our cohort. Additionally, our study was the first to analyze the effectiveness of US AI DS in detecting breast sarcomas. AI DS has been shown to be useful in detecting breast cancers (with a sensitivity of 98%), and we hypothesized that it could be useful in the sarcoma population [30].

## 2. Methods

### 2.1. Data Acquisition and Patients

An IRB-approved, retrospective review from 2008–2024 identified all patients over 18 years of age with biopsy-proven breast sarcomas in our radiology database, which we queried using the term “breast sarcoma”. Consecutive sampling was performed to mitigate selection bias. Informed consent was waived by the IRB. Exclusion criteria included a lack of imaging prior to resection; every case that had imaging prior to resection was included. No image preprocessing was performed prior to AI analysis. Some cases included recurrent lesions, which were included in our study. Clinical information including age, gender, clinical presentation, and precise histopathological diagnosis was accumulated in an HIPAA-compliant digital format.

### 2.2. Imaging

Mammography consisted of at least standard mediolateral oblique (MLO) and cranio-caudal (CC) images. Mammography examinations from our institution were performed on Hologic Selenia (Hologic Inc., Danbury, CT, USA). There were 5 mammograms performed at outside facilities that were submitted for review by an internal radiologist.

Ultrasound imaging at our institution was performed by certified ultrasound technologists using a hand-held linear transducer at a center frequency of 10–18 MHz on IU22 (Philips, Cambridge, MA, USA) or Epiq Elite (Philips, Cambridge, MA, USA). Grey-scale and color Doppler images were obtained for each ultrasound lesion in radial and anti-radial views. There were 10 ultrasounds performed at an outside facility that were submitted for review by an internal radiologist.

Magnetic resonance imaging (MRI) of the breast with contrast at our institution was performed using a dedicated breast coil on one of the following scanners: 3T GE Discovery MR750 (GE Healthcare Technologies Inc., Chicago, IL, USA), 1.5T GE Signa (GE Healthcare Technologies Inc., Chicago, IL, USA), or 1.5T Siemens Magnetom Sola (Siemens Healthineers, Erlangen, Germany). Sequences included the following: axial T1-weighted and T2 STIR sequences, dynamic pre- and post-contrast fat-saturated axial images after intravenous injection of Gadavist contrast, along with delayed post-contrast sagittal fat-saturated T1-weighted images. There was one MRI exam performed at an outside facility that was submitted for review by an internal radiologist.

### 2.3. Imaging Analysis

All available images were retrospectively reviewed by two non-blinded radiologists (DW and CC) with 8 years of experience each on the Picture Archiving and Communication System (PACS). Imaging features on mammography, ultrasound, and MRI were classified according to the Breast Imaging Reporting and Data Systems (BI-RADS) 5th edition lexicon [31]. Lesion size was determined by measuring the maximum diameter. Readings were independent prior to consensus, and 100% inter-reader agreement was obtained for the BI-RADS lexicon imaging features of the identified breast lesions. Imaging with “no suspicious findings” was defined as a truly negative study.

Measurements were obtained based on ultrasound dimensions, with the largest dimension recorded for each lesion. For patients with skin thickening in the absence of a lesion, no lesion size was obtained. Patients with distortion on mammography without a discrete mass on ultrasound also did not have measurements included in summary statistics. Ultrasound-based measurements were selected over mammographic or MRI measurements, as ultrasound data were available for all cases.

### 2.4. AI Decision Support

KOIOS AI DS (KOIOS Medical Inc., version 3.6.1, New York, NY, USA) was used retrospectively on breast lesions seen on ultrasound, whether the exam was performed at our institution or elsewhere. A single region of interest (ROI) was manually placed by a radiologist (CC) over the biopsy-proven breast sarcoma lesions in orthogonal views (i.e., radial and anti-radial) on pre-biopsy b-mode, grey-scale images. AI DS generated a probability of malignancy that was stratified into four categories and aligned with likelihood of malignancy (LOM) and the BI-RADS categories: benign (LOM < 0.5%, BI-RADS 2), probably benign (LOM < 2%, BI-RADS 3), suspicious (LOM < 50%, BI-RADS 4A or 4B), and probably malignant (LOM > 50%, BI-RADS 4C or 5) [32]. The a priori primary endpoint was the sensitivity to detect suspicious lesions warranting biopsy.

### 2.5. Statistical Analysis

Data analysis was performed using descriptive statistics. Continuous variables were expressed as mean ± standard deviation (SD) and categorical variables as percentages with a 95% confidence interval included in brackets.

## 3. Results

### 3.1. Diagnosis and Clinical Presentation

Retrospective review of our radiology database identified 22 patients with breast sarcoma. A total of 4 of the 22 patients were excluded due to a lack of imaging available prior to resection, leaving 18 patients in the final cohort. Of the 18 patients, there were 9 with PBS and 9 with SBS. A total of 11% (2/18) [0.01–0.26] of patients included in the study experienced disease recurrence, one with PBS and one with SBS. Patient demographics and pathologic and clinical presentation of the breast sarcomas in our cohort are shown in Table 1.

Seventeen female patients (mean age 53.9 years ± 20.8; range 20–80 years) and one male patient (age 72 years) were included in the study. A total of 9/18 patients (50%) [0.26–0.74] presented with a palpable lump, 5/18 (27.8%) [0.10–0.53] presented with breast skin changes, 2/18 (11.1%) [0.01–0.35] presented with a palpable lump with associated overlying skin changes, 1/18 (5.6%) [0.001–0.27] presented with asymmetric breast enlargement, and 1/18 (5.6%) [0.001–0.27] presented with increased nipple sensitivity. In all 18 patients (100%) [0.81–1.00], the method of detection was “P—Patient detected by self-exam or symptom, or Provider detected with clinical exam”, meaning that the disease was first detected either by the patient via self-exam or symptom OR was detected by the provider on clinical exam. None were detected during routine screening. A total of 2/18 (11.1%) [0.01–0.35] patients were pregnant at the time of diagnosis. For patients with SBS, the median latency between radiation and sarcoma development was 6.5 months (±4.4 months, range 4–13 months).

Radiologic features of breast sarcomas are shown at the case level in Table 2. A total of 15/18 (83%) [0.59–0.96] patients had an identifiable lesion on at least one imaging modality, and 3/18 (17%) [0.04–0.41] patients had skin thickening only without an identifiable intra-mammary finding on imaging. The average lesion size was 2.6 cm by imaging (range 0.5 cm–7.1 cm; 95% CI [1.69–3.51]). Two out of eighteen patients (11.1%) [0.01–0.35] included in the study experienced disease recurrence. Both cases of recurrence presented as palpable lumps; one recurrence occurred in the contralateral breast diagnosed 22 months after initial diagnosis, while a second case occurred in the ipsilateral breast 12 months after initial diagnosis.

The most commonly diagnosed sarcomas in our study were angiosarcoma (13/18 [72.2%]) [0.47–0.90], followed by spindle cell sarcoma (2/18 [11.1%]) [0.01–0.35]. Other histopathological subtypes of breast sarcomas included granulocytic sarcoma, high-grade pleomorphic sarcoma, and fibromyxoid sarcoma (1/18 [5.6%] [0.001–0.27] each, respectively, Table 2).

### 3.2. Mammographic Findings

**Figure 1:**
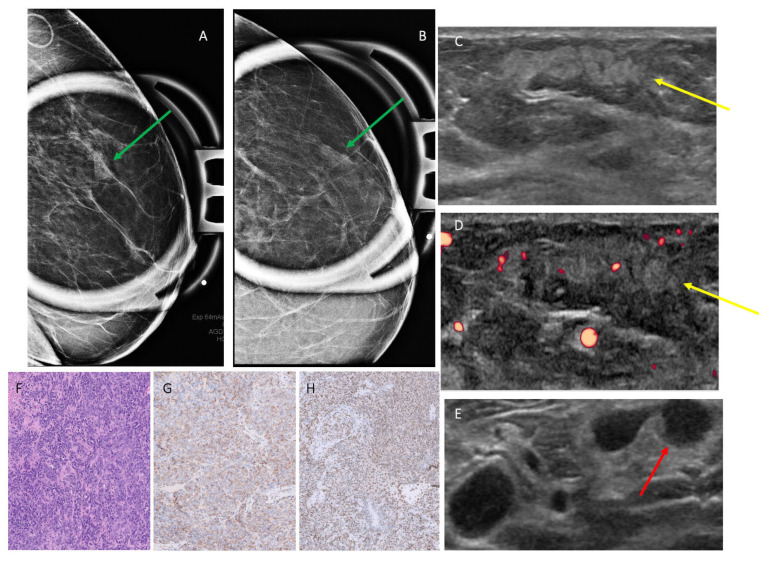
Patient 12 in Table 1 and Table 2, a 79-year-old woman with history of left breast cancer status after lumpectomy and chemoradiation, presenting with skin thickening and a palpable lump. (**A**,**B**) Mammogram obtained, with green arrows highlighting a focal asymmetry on CC and MLO spot compression views. (**C**,**D**) Ultrasound targeted to the area of palpable finding, with yellow arrow showing a corresponding 2.9 cm parallel, irregular hyperechoic mass with obscured margins and vascular flow, along with (**E**) a red arrow pointing to an abnormal lymph node with cortical thickening. Subsequent ultrasound-guided biopsy yielded angiosarcoma. (**F**) H&E staining of a solid and spindled growth pattern with marked atypia representing a high-grade tumor. (**G**,**H**) Immunohistochemistry showed positivity for CD31 and FLI-1, respectively, while epithelial markers were negative, indicating a tumor of vascular origin. AI DS = suspicious, 4A–4B.

Thirteen cases were imaged with mammography. A total of 3/13 cases (23.1%) [0.05–0.54] presented as an irregular, indistinct, equal, or high-density mass (Figure 1). A total of 3/13 cases (23.1%) [0.05–0.54] presented as a focal or global asymmetry without calcifications, 1/13 cases (7.7%) [0.002–0.36] presented as architectural distortion, and 1/13 cases (7.7%) [0.002–0.36] presented as asymmetry with amorphous calcifications. A total of 2/13 cases (15.4%) [0.02–0.45] had no significant mammographic findings, and 3/13 cases (23.1%) [0.05–0.54] presented as skin thickening only without other associated intra-mammary findings (Table 2).

### 3.3. Ultrasound Findings

**Figure 2:**
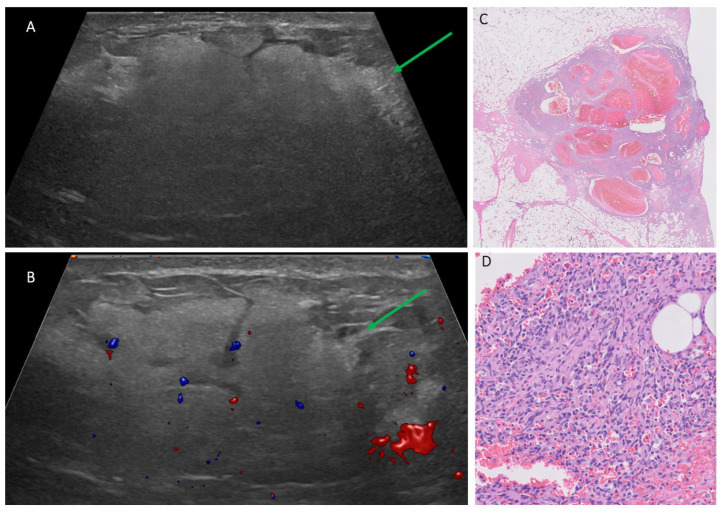
Patient 14 in Table 1 and Table 2, a 22-year-old pregnant female with a growing left breast lump. (**A**,**B**) Targeted ultrasound to the palpable area, with green arrows pointing to a 7.1 cm parallel, mixed-echogenicity irregular lesion between 1:00–3:00, 6 cm from the nipple, with obscured margins and vascular flow. Ultrasound-guided biopsy yielded high-grade angiosarcoma. (**C**) Histopathology with H&E stain showed a vascular proliferation. (**D**) High-power view revealed solid areas of spindled tumor cells with cytologic atypia and increased mitotic activity. Immunohistochemistry further confirmed vascular origin, supporting a diagnosis of high-grade angiosarcoma. AI DS = suspicious, 4A–4B.

**Figure 3:**
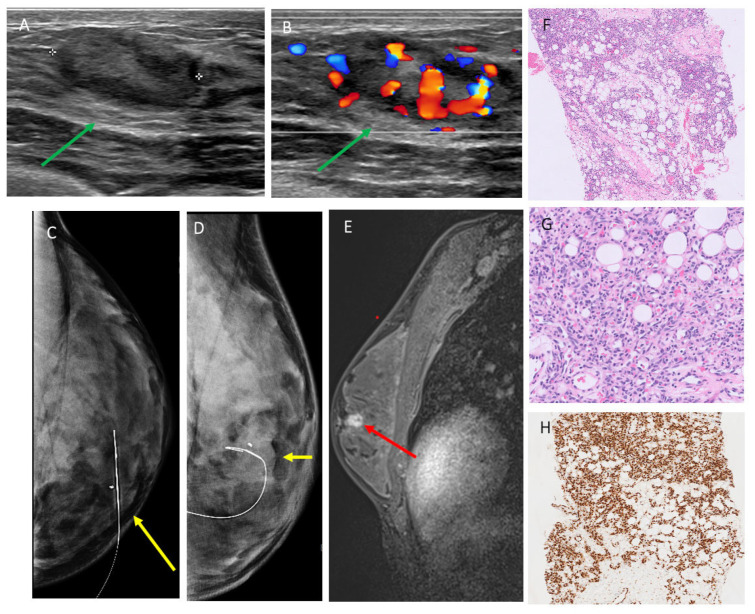
Patient 9 in Table 1 and Table 2, a 25-year-old woman presented with left breast lump for 3 months. (**A**,**B**) Ultrasound targeted to the palpable lump shows a 2.5 cm highly vascular, mixed-echogenicity mass in the left breast 9:00, 2 cm from the nipple, highlighted by green arrows. Subsequent ultrasound-guided biopsy yielded angiosarcoma. (**C**,**D**) Mammogram obtained after wire localization showing the biopsied mass, highlighted by yellow arrows, with clip adjacent to the reinforced segment of the wire on CC and ML views, respectively. (**E**) Contrast-enhanced MRI demonstrated a corresponding irregular heterogeneously enhancing mass (red arrow). (**F**,**G**) H&E stains showing proliferation of vascular channels with hyperchromatic endothelial cells dissecting through fat. (**H**) Immunohistochemistry showing diffuse positivity for vascular marker ERG. AI DS = suspicious, 4A–4B.

**Figure 4:**
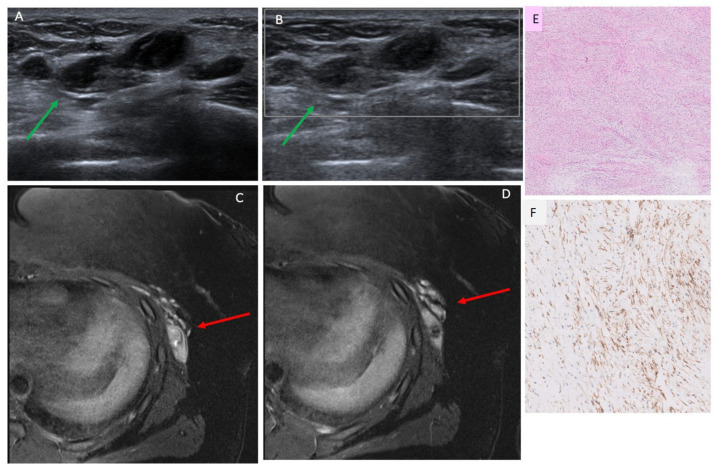
Patient 5 in Table 1 and Table 2, a 20-year-old woman with new palpable lump in the left lateral chest wall. (**A**,**B**) Ultrasound targeted to the palpable lump showed a 3.4 cm irregular hypoechoic mass located in the left lateral breast, 17 cm (green arrow), which had posterior acoustic enhancement and absence of vascular flow. Biopsy yielded fibromyxoid sarcoma. (**C**,**D**) Contrast-enhanced MRI show a corresponding lobulated, irregular mass with heterogenous enhancement, highlighted by red arrows. (**E**) H&E stain showing a bland spindle cell neoplasm with low-to-moderate cellularity and myxoid background. (**F**) Immunohistochemistry demonstrating MUC4 positivity, supporting the diagnosis of low-grade fibromyxoid sarcoma. AI DS = suspicious, 4A–4B.

The ultrasound features of 19 breast sarcoma cases (including 2 recurrent cases) in our cohort are shown in Table 2. A total of 16/19 cases (84.2%) [0.60–0.97] presented with a mass, 1/19 case (5.2%) [0.001–0.26] presented with only skin thickening, and no suspicious sonographic finding was identified in 2/19 (10.5%) [0.01–0.33] cases. Most masses were irregular in shape (13/16 [81.2%] [0.01–0.33]), followed by oval in shape (3/16 [18.8%] [0.03–0.40]), and they were commonly parallel in orientation (14/16 [87.5%] [0.49–0.91]) with non-circumscribed margins (15/16 [93.7%] [0.54–0.94]). Echo pattern was variable (Figure 2, Figure 3 and Figure 4), with hypoechoic being the most common (10/16 [62.5%] [0.35–0.85]), followed by heterogenous (5/16 [31.2%] [0.11–0.59]) and hyperechoic echo patterns (1/16 [6.2%] [0.002–0.30]). The majority of masses demonstrated posterior acoustic enhancement (12/16 [75%] [0.48–0.93]) and internal vascularity (12/16 [75%] [0.48–0.93]).

### 3.4. MRI Findings

Nine cases in our cohort had MRIs available for review. A total of 6/9 (66.7%) [0.30–0.93] presented as an intramammary mass, most commonly with irregular margins and heterogenous enhancement (Figure 3 and Figure 4). A total of 3/9 (33.3%) [0.07–0.70] presented as skin thickening and enhancement without an associated intramammary mass on MRI (Table 2).

### 3.5. AI Analysis

AI DS assessment was available in 16 ultrasound cases with breast sarcoma. In the majority of cases, AI DS identified breast sarcoma lesions as suspicious (14/16 cases [87.5%] [0.62–0.98]) or probably malignant (1/16 [6.3%] [0.002–0.30]). AI DS misclassified one case (6.3%) [0.002–0.30], identifying it as probably benign. AI DS was not able to generate a result for cases where only skin thickening was present but where there was no intramammary mass (Table 2).

## 4. Discussion

This retrospective imaging case series—spanning one of the longest durations to date and comprising one of the largest recent sample sizes—uniquely highlights the broader-than-expected imaging variability in breast sarcomas across ultrasound, mammography, and MRI, while also incorporating AI-driven decision support to enhance diagnostic assessment [5,6,33]. Our study utilized multi-modality imaging, including mammography, ultrasound, and MRI, to assess and describe the variable features of breast sarcomas. In addition to the variable imaging presentation of sarcomas, we highlighted rare clinical presentations of breast sarcomas as well, including occurrence in a male patient, diagnosis during pregnancy, and tumor recurrence. Sarcomas are exceedingly rare in the male breast, accounting for 1.5% of breast sarcomas altogether, with only sporadic reports in the literature of breast sarcoma in men [17]. Similarly, sarcoma diagnosed during pregnancy is a rare presentation, with only several case reports in the literature. At this time, there is no established relationship or known risk factors between pregnancy and breast sarcoma. It is suggested that 6–12% of primary breast angiosarcomas are diagnosed during pregnancy or shortly after, suggesting hormone involvement, although hormone receptor positivity is rare, making it impossible to assign a link between estrogen dependency and angiosarcoma [34]. The nonspecific clinical and radiologic presentation of breast sarcomas, along with pregnancy-related changes in breast tissue, can provide further diagnostic challenges to clinicians and radiologists. Two patients with local recurrence were seen in our study population, which is consistent with the aggressive nature of the disease, with a rate of recurrence as high as up to 73% of cases [12].

Breast sarcomas typically occur without associated skin findings [17]. However, three patients in our cohort presented with skin thickening only without breast findings. This presentation can be a diagnostic challenge and can lead to a delay in treatment if appropriate clinical follow-up is not pursued after imaging. In our cohort, the three patients with this presentation all had a prior history of radiation therapy to the breast and were ultimately diagnosed with secondary breast sarcoma—all of which were of the angiosarcoma subtype. This is similar to Smith et al.’s study, which described two cases in their cohort that only presented with skin thickening and were subsequently found to have radiation-associated angiosarcoma of the breast [6]. Our study helps to further highlight the importance of clinical history taking and to raise suspicion in cases of isolated skin thickening in patients with a history of radiation and to recommend further workup, including punch biopsy.

In our cohort, mammographic presentations of breast sarcomas ranged from the presence of an indistinct irregular mass, focal or global asymmetry, focal asymmetry with calcifications, architectural distortion, skin thickening in the absence of other findings, or a negative mammogram. The imaging distribution observed in our study was notably more variable than previously reported, where breast sarcomas have typically been described as either well-circumscribed, oval-shaped masses or areas of architectural distortion [5,6,7]. These traditional patterns have shaped expectations and may contribute to missed or delayed diagnoses in atypical cases. We also observed a rare case of a focal asymmetry with amorphous calcifications, consistent with emerging data showing that up to 10% of breast sarcomas may present with calcifications [3]. These unique findings expand the known imaging spectrum of breast sarcomas and highlight the need for increased awareness of their variable appearances across imaging modalities.

On ultrasound, most breast masses in our cohort were irregular in shape, with hypoechoic echogenicity, non-circumscribed margins, and posterior acoustic enhancement. Although our findings regarding lesion shape are consistent with those reported by Wienbeck et al., they contrast with the majority of the existing literature, which has typically described breast sarcomas as oval or round in shape with circumscribed margins [6,7,31,32,33]. Internal vascularity is a feature that has been infrequently discussed in the literature; however, studies that do address it consistently report it as a predominant finding—consistent with our cohort [6,12,17]. Additionally, most of the masses from our cohort demonstrated posterior acoustic enhancement—a feature that has been reported in several previous studies [6,7,12]. However, Weinbeck et at. and Matsumoto et al. have described posterior acoustic shadowing as the predominating finding in their studies, further highlighting the highly variable imaging characteristics of breast sarcoma [17,33]. Overall, our data reveal a distinct ultrasound profile that diverges from traditional imaging expectations. There were several lesions that also demonstrated heterogeneous or hyperechoic echogenicity, which can be subtle on imaging. This less common and more challenging presentation has also been described in the literature, at rates of up to 37% in some studies [5,6,7,9]. In our cohort, no breast sarcomas were identified through routine screening, and 6 of 18 cases (33.3%) occurred in patients aged 40 years or younger. These findings underscore a potential gap in current breast cancer screening guidelines. Notably, ultrasound demonstrated strong diagnostic performance in our study and is a safe, widely accessible imaging modality, suggesting that it may have value as a screening tool for younger women who are not currently covered by standard screening recommendations.

Our cohort included nine patients with MRI imaging available, with the majority of cases comprising of irregular-shaped masses with irregular margins and heterogeneous enhancement. The findings regarding shape and margins are concordant with the study by Wienbeck et al.; however, they differ slightly from the study by Smith et al., where the morphology was more commonly described as a round or oval mass with irregular margins [6,33]. A portion of our cases presented only with skin thickening and enhancement on MRI and no associated breast mass. This finding also highlights the importance of being aware of the broad imaging findings of breast sarcomas along with radiologic–pathologic correlation in making the diagnosis of breast sarcomas.

**Figure 5:**
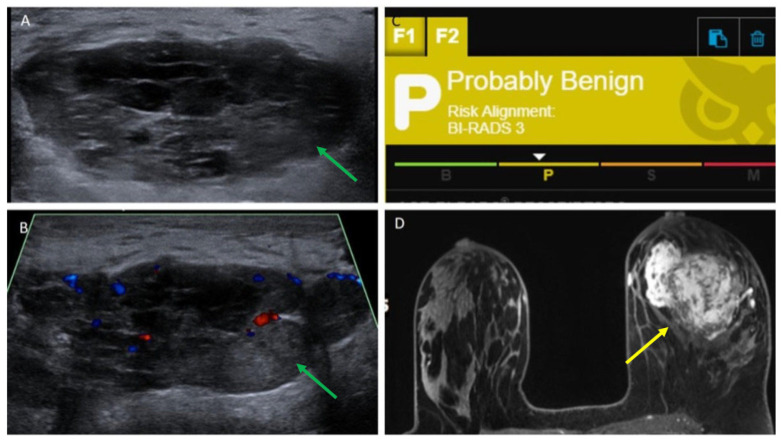
Patient 16 in Table 1 and Table 2, a 29-year-old woman with new palpable lump in the left breast. (**A**,**B**) Ultrasound targeted to the palpable lump showed a 5.5 cm oval, circumscribed, parallel hypoechoic mass (shown by green arrows) with mixed solid and cystic components, as well as internal vascularity. (**C**) AI DS assessment of probably benign was obtained. (**D**) Contrast-enhanced MRI showed a corresponding lobulated, irregular mass (yellow arrow) with heterogenous enhancement. Ultrasound-guided biopsy yielded angiosarcoma. The lack of color Doppler evaluation by AI DS may have contributed to the incorrect “probably benign” assessment.

Our study is the first to evaluate the use of ultrasound AI DS in detecting breast sarcomas. In the majority of cases (93.8%), AI DS categorized breast sarcoma lesions as suspicious or probably malignant, which appears comparable to sensitivities reported in other studies evaluating AI DS for breast carcinomas, which range from 0.95 to 0.98 [28,29]. AI DS misclassified one case, identifying it as probably benign. Interestingly, this lesion presented similarly to a complex cystic lesion on grey-scale imaging (Figure 5). This finding is consistent with reports in the literature describing rare instances where breast sarcomas have mimicked cystic lesions on imaging, often due to their heterogeneous internal architecture or necrotic components [35,36]. At present, AI DS does not analyze Doppler flow within lesions. When assessing cystic masses on ultrasound in patients with suspected breast sarcoma based on clinical presentation, extra caution is necessary. Radiologists should be careful not to rely solely on AI-based tools, recognizing that AI DS is intended to assist clinical decision-making rather than replace it. Ultimately, the radiologist’s expertise and judgment should take precedence over AI DS evaluations when needed. Future research and software enhancements are essential to better detect solid masses that appear cystic and to integrate Doppler flow analysis, which plays a vital role in breast imaging. Additionally, our AI DS analysis was limited to ultrasound only and we did not include assessment of any AI DS for breast MRI or mammography. AI DS was also not able to generate a result for cases where only skin thickening was present but no mass. This is a limitation of AI DS, given that breast sarcomas can present with only skin findings such as skin thickening or rash and no palpable mass. Also, our AI DS assessment is a descriptive, proof-of-concept sensitivity estimate, and this study is a malignancy-enriched cohort. Therefore, specificity, Area Under the Curve (AUC), and overall diagnostic accuracy could not be assessed.

The KOIOS algorithm was trained on a large, diverse dataset of over 450,000 breast ultrasound images and breast lesions with known pathologic outcomes (cite) across over 30 clinical sites and 40 models of ultrasound machines from all major manufacturers. The dataset includes a broad spectrum of malignant and benign breast lesions. However, details regarding the full histopathologic breakdown of the training cases by subtype that includes or excludes sarcoma by name is not available, and therefore, it is unknown if any breast sarcoma cases were utilized. Although KOIOS DS is not indicated for the diagnosis of sarcoma, three sarcoma cases were included in the training dataset [30,37]. Nonetheless, our study showed that KOIOS AI DS performed well in assigning the majority of sarcoma lesions as suspicious or probably malignant.

Limitations to our study include the retrospective nature of the study and a small sample size, which limits comparative evaluation of PBS and SBS. Previous studies have demonstrated that PBS has a younger mean age at diagnosis compared to SBS and more commonly presents as a palpable mass. Alternatively, SBS is more frequently associated with a history of prior breast radiation and often of the angiosarcoma subtype and has a higher occurrence of presenting as overlying skin changes with or without an underlying mass. Although our cohort does display similarities with studies that have compared PBS versus SBS, our small sample size limits us from making a true comparative assessment and conclusion [14,20].

Few patients in our cohort underwent imaging with all the modalities of mammography, ultrasound, and MRI, although this reflects typical clinical practice. We included imaging from outside institutions, leading to a wide variety of mammogram and ultrasound machines used with decreased standardization of technique and image acquisition. Additionally, readings were non-blinded.

Regarding the use of AI DS, the AI analysis is post hoc with manual ROI placement by a radiologist retrospectively on selected B-mode images. This process can introduce incorporation and selection biases. However, the retrospective ROI placement is less likely to influence model predictions, as determined by Barinov et al., where the KOIOS category shifted minimally between “probably benign” and “suspicious” classifications when ROI boundaries are varied, suggesting a limited impact on overall model output [38]. The use of a single-vendor AI system and absence of benign comparators are additional limitations in this study. Future direction could include an analysis of cases with different AI vendors, including AI DS for mammography and MRI, where features unique to those imaging modalities, such as MRI kinetics, can also be utilized in determining whether a mass is suspicious or not.

Our study has shown that breast sarcomas have a variable clinical and radiologic presentation which can result in challenges for clinicians and radiologists alike. On mammography, most breast sarcomas in our cohort either presented as a focal asymmetry or as a high-density mass with irregular shape and indistinct margins. On ultrasound, most breast masses were irregular in shape, with hypoechoic echogenicity, obscured or indistinct margins, and posterior acoustic enhancement. These findings are somewhat discordant with the majority of the existing literature, suggesting that there are few, if any, reliable findings on imaging to be considered pathognomonic for breast sarcomas. Interestingly, skin thickening was noted across all three modalities, suggesting that clinicians should include breast sarcomas in their differential diagnosis for patients who present primarily with skin findings, particularly in the setting of prior radiation therapy. AI successfully classified nearly all breast sarcoma lesions as suspicious for malignancy or probably malignant for the cases in our study. However, the absence of a discrete mass in certain cases of breast sarcoma may limit the use of available AI software in detecting suspicious masses. An additional limitation of this study is that AI performance was assessed within a malignancy-only cohort in a feasibility observation, which limits evaluation of its general diagnostic accuracy.

## 5. Conclusions

Breast sarcomas are rare and aggressive. Awareness of how breast sarcomas can present across imaging modalities, while using AI DS as an aid, may help radiologists in making the correct diagnosis of this rare and aggressive disease.

## Figures and Tables

**Table 1:** Patient demographics and pathologic and clinical presentation of breast sarcomas.

PT	Age	Gender	PBS vs. SBS	Prior RT?	SBS Latency from RT (Months)	Clinical Manifestation	Histopathologic Subtype/Grade	IHC Markers	Recurrence?
1	67	F	SBS	Y	13	Palpable lump	Spindle Cell/High	P63+, ER−, PR−, Her2−	N
2	22	F	PBS	N	N/a	Asymmetric breast enlargement	Granulocytic/N/a	CD68+, CD34+, MPO+, lysozyme+, CD43+, CD117+	N
3	72	M	PBS	N	N/a	Increased nipple sensitivity	Pleomorphic/High	Desmin+, scattered stromal cells ER+ and PR+, CD163+, SMA−, myoD−, Myf4	N
4	57	F	PBS	N	N/a	Palpable lump	Spindle Cell/High	ER−, PR-Her2−,	N
5	20	F	PBS	N	N/a	Palpable lump	Fibromyxoid/Low	FXIII+, BCL-2+	N
6	65	F	SBS	Y	5	Skin changes	Angiosarcoma/High	CD31+, CD34+, ERG+, C-MYC+, ER−, PR−, Her-2−	N
7	74	F	SBS	Y	6	Skin changes	Angiosarcoma/N/a	Ki67+, MYC+,	N
8	80	F	SBS	Y	5	Palpable lump	Angiosarcoma/High	N/a	N
9	25	F	PBS	N	N/a	Palpable lump	Angiosarcoma/Low–intermediate	N/a	N
10	69	F	PBS	N	N/a	Palpable lump	Angiosarcoma/N/a	N/a	N
11	53	F	SBS	Y	8	Skin changes	Angiosarcoma/N/a	ERG+, CD34+, C-MYC−	N
12	79	F	SBS	Y	18	Skin changes	Angiosarcoma/High	CD31+, C-MYC+, FLI1+	N
13	72	F	SBS	Y	7	Palpable lump	Angiosarcoma/Low	N/a	N
14	22	F	PBS	N	N/a	Palpable lump	Angiosarcoma/High	ERG+, CD31+, Ki67+ (50–60%)	N
15a	40	F	PBS	N	N/a	Palpable lump with overlying skin changes	Angiosarcoma/Intermediate–High	N/a	N
15b	41	F	PBS	N	N/a	Palpable lump	Angiosarcoma/Intermediate–High	N/a	Y
16	29	F	PBS	N	N/a	Palpable lump	Angiosarcoma/High	Ki67+ (60%)	N
17a	61	F	SBS	Y	4	Palpable lump with overlying skin changes	Angiosarcoma/High	N/a	N
17b	62	F	SBS	Y	5	Palpable lump	Angiosarcoma/High	ERG+, CD31+, FVIII+	Y
18	68	F	SBS	Y	8	Skin changes	Angiosarcoma/High	MYC+, ERG+. Ki67+ (75%), CD34+, F8+	N

Key: PT = Patient; PBS = primary breast sarcoma; SBS = secondary breast sarcoma; RT = radiation; Y = yes; N = no; IHC = immunohistochemistry; N/a = not available.

**Table 2:** Radiologic features of breast sarcomas.

Patient	Size (cm)	Biopsy Method	MG Features	US Features	AI DS	MRI Features
1	1.8	Ultrasound core needle biopsy	Irregular high-density mass with indistinct margins	Irregular, non-parallel hypoechoic mass with microlobulated margins, posterior acoustic enhancement and no Doppler flow	Suspicious, 4A–4B	Irregular mass with irregular margins and heterogeneous enhancement
2	1.4	Ultrasound core needle biopsy	Global asymmetry	Irregular, non-parallel, heterogeneous mass with indistinct margins, posterior acoustic shadowing and Doppler flow	Suspicious, 4A–4B	N/a
3	0.9	Ultrasound core needle biopsy	Gynecomastia, otherwise no suspicious findings	Oval, parallel, hypoechoic mass with circumscribed margins, posterior acoustic enhancement and no Doppler flow	Suspicious, 4A–4B	N/a
4	3.5	Ultrasound core needle biopsy	Focal asymmetry associated with amorphous calcifications	Irregular, parallel, hypoechoic mass with indistinct margins, posterior acoustic shadowing and Doppler flow	Probably Malignant, 4C	N/a
5	3.4	Ultrasound core needle biopsy	N/a	Irregular, parallel, hypoechoic masses with indistinct/angular margins, posterior acoustic enhancement and no Doppler flow	Suspicious, 4A–4B	Lobulated mass with irregular margins and heterogeneous enhancement
6	N/a	Skin punch biopsy	Skin thickening only	N/a	N/a	N/a
7	2.7	Skin punch biopsy	N/a	Irregular, parallel, hypoechoic mass with microlobulated margins, posterior acoustic enhancement and Doppler flow	Suspicious, 4A–4B	N/a
8	N/a	Skin punch biopsy	Architectural distortion	Irregular skin thickening only	N/a	Diffuse irregular skin thickening of almost entire right breast with discontinuous areas of linear and nodular enhancement within the thickened skin
9	2.5	Ultrasound core needle biopsy	N/a	Oval, parallel, heterogeneous mass with indistinct margins, posterior acoustic enhancement and Doppler flow	Suspicious, 4A–4B	Irregular mass with irregular margins and heterogeneous enhancement
10	2.5	Ultrasound core needle biopsy	Irregular high-density mass with indistinct margins	Irregular, parallel, heterogeneous mass with indistinct margins, posterior acoustic enhancement and Doppler flow	Suspicious, 4A–4B	Irregular mass with irregular margins and heterogeneous enhancement
11	N/a	Skin punch biopsy	No suspicious findings	No suspicious findings	N/a	Skin thickening and enhancement
12	2.9	Ultrasound core needle biopsy	Focal asymmetry	Irregular, parallel, hyperechoic mass with obscured margins, posterior acoustic enhancement and Doppler flow	Suspicious, 4A–4B	N/a
13	0.8	Skin punch biopsy	N/a	Irregular, parallel, heterogeneous mass with obscured margins, posterior acoustic shadowing and no Doppler flow	Suspicious, 4A–4B	N/a
14	7.1	Ultrasound core needle biopsy	N/a	Irregular, parallel, heterogeneous mass with obscured margins, posterior acoustic enhancement and Doppler flow	Suspicious, 4A–4B	N/a
15a	3.7	Ultrasound core needle biopsy	N/a	Irregular, parallel, heterogeneous mass with obscured margins, posterior acoustic shadowing and Doppler flow	Suspicious, 4A–4B	N/a
15b	0.5	Ultrasound core needle biopsy	Focal asymmetry	Irregular, parallel, hypoechoic mass with obscured margins, posterior acoustic enhancement and Doppler flow	Suspicious, 4A–4B	Irregular mass with irregular margins and skin enhancement
16	5.5	Ultrasound core needle biopsy	Irregular, equal-density mass with indistinct margins	Oval, parallel, hypoechoic mass with obscured margins, posterior acoustic enhancement and Doppler flow	Probably Benign, 3	Irregular mass with irregular margins and heterogeneous enhancement
17a	1.2	Ultrasound core needle biopsy	Skin thickening only	Irregular, parallel, hypoechoic mass with obscured margins, posterior acoustic enhancement and Doppler flow	Suspicious, 4A–4B	N/a
17b	1.7	Ultrasound core needle biopsy	N/a	Irregular, parallel, hypoechoic mass with indistinct margins, posterior acoustic enhancement and no Doppler flow	Suspicious, 4A–4B	N/a
18	N/a	Skin punch biopsy	Skin thickening only	No suspicious finding	N/a	Irregular skin thickening of the left breast with heterogeneously enhancing skin lesion

Key: MG = mammogram; US = ultrasound; AI DS = artificial intelligence decision support, MRI = magnetic resonance imaging; N/a = not available.

## Data Availability

The original contributions presented in this study are included in the article. Further inquiries can be directed to the corresponding authors.
